# Cytological Profile of Antibacterial FtsZ Inhibitors and Synthetic Peptide MciZ

**DOI:** 10.3389/fmicb.2016.01558

**Published:** 2016-10-03

**Authors:** Lidia Araújo-Bazán, Laura B. Ruiz-Avila, David Andreu, Sonia Huecas, José M. Andreu

**Affiliations:** ^1^Centro de Investigaciones Biológicas, Consejo Superior de Investigaciones CientíficasMadrid, Spain; ^2^Department of Experimental and Health Sciences, Universitat Pompeu FabraBarcelona, Spain

**Keywords:** bacterial cell division, FtsZ ring, small molecule inhibitors, MciZ, cytological profiling

## Abstract

Cell division protein FtsZ is the organizer of the cytokinetic ring in almost all bacteria and a target for the discovery of new antibacterial agents that are needed to counter widespread antibiotic resistance. Bacterial cytological profiling, using quantitative microscopy, is a powerful approach for identifying the mechanism of action of antibacterial molecules affecting different cellular pathways. We have determined the cytological profile on *Bacillus subtilis* cells of a selection of small molecule inhibitors targeting FtsZ on different binding sites. FtsZ inhibitors lead to long undivided cells, impair the normal assembly of FtsZ into the midcell Z-rings, induce aberrant ring distributions, punctate FtsZ *foci*, membrane spots and also modify nucleoid length. Quantitative analysis of cell and nucleoid length combined, or the Z-ring distribution, allows categorizing FtsZ inhibitors and to distinguish them from antibiotics with other mechanisms of action, which should be useful for identifying new antibacterial FtsZ inhibitors. Biochemical assays of FtsZ polymerization and GTPase activity combined explain the cellular effects of the FtsZ polymer stabilizing agent PC190723 and its fragments. MciZ is a 40-aminoacid endogenous inhibitor of cell division normally expressed during sporulation in *B. subtilis*. Using FtsZ cytological profiling we have determined that exogenous synthetic MciZ is an effective inhibitor of *B. subtilis* cell division, Z-ring formation and localization. This finding supports our cell-based approach to screen for FtsZ inhibitors and opens new possibilities for peptide inhibitors of bacterial cell division.

## Introduction

Cell division protein FtsZ, a tubulin-like GTPase conserved in most bacteria, is a target for new antibiotics. At the earliest step of cell division, FtsZ undergoes assembly at mid-cell forming a dynamic membrane-attached ring structure (Bi and Lutkenhaus, 1991). Other bacterial division proteins are then recruited to this Z-ring to form the divisome, a complex that constricts between the future daughter cells (Adams and Errington, 2009; Lutkenhaus et al., 2012; Egan and Vollmer, 2013; Meier and Goley, 2014; Haeusser and Margolin, 2016). FtsZ assembles into polar tubulin-like protofilaments in which the GTP-binding site of one monomer is at the association interface with the next monomer completing the GTPase site (Oliva et al., 2004; Matsui et al., 2012). Dynamic FtsZ filaments laterally associate in different fashions including double filaments, bundles, and ribbons (Erickson et al., 2010; Buske and Levin, 2012, Buske et al., 2015). Electron cryotomography studies have shown a small band of individual, laterally connected FtsZ filaments forming a ring parallel to the membrane (Szwedziak et al., 2014). However, super resolution fluorescence microscopy in different organisms suggests that the Z-ring is a patchy scaffold made of disordered FtsZ protofilaments (Fu et al., 2010; Biteen et al., 2012; Strauss et al., 2012; Si et al., 2013; Holden et al., 2014; Rowlett and Margolin, 2014). The Z-ring is stabilized by a protein network connecting the cell membrane to the chromosome in *Escherichia coli* cells (Buss et al., 2015), where the constriction force has been suggested to come mainly from the septal cell wall synthesis (Coltharp et al., 2016).

The functional inhibition of FtsZ blocks cell division and induces the formation of long, multi-nucleoid cell filaments via uncoupling growth and division. Mutations in *ftsZ* affect cell division, FtsZ polymerization and GTPase activity (Stricker and Erickson, 2003; Feucht and Errington, 2005; Redick et al., 2005). Several protein inhibitors and physiological mechanisms inhibit FtsZ directly and block cell division (Margolin, 2005). For example, DNA damage initiates the SOS response and triggers expression of SulA, which blocks the FtsZ minus-end for assembly and stalls division for DNA damage repair (Bi and Lutkenhaus, 1993; Cordell et al., 2003). Another mechanism is provided by the nucleoid occlusion machinery that prevents the assembly of functional Z-rings in membrane areas in close proximity to the nucleoid (Bernhardt and de Boer, 2005; Wu et al., 2009; Adams et al., 2015). The loss of the transmembrane potential has also been reported to negatively affect FtsZ function, inhibiting cell division via dissociation of FtsA and consequent release of FtsZ from the membrane in *Bacillus subtilis* (Strahl and Hamoen, 2010). Recently, a link of cell division to central carbon metabolism has been established, including the discoveries that UDP-glucose-activated UgtP and OpgH enzymes inhibit FtsZ assembly until cells reach an appropriate mass (Hill et al., 2013), and that pyruvate may promote Z-ring assembly via PDH E1α (Monahan et al., 2014). On the other hand, the developmental regulator MciZ (mother cell inhibitor of Z), a 40-amino acid peptide produced during *B. subtilis* sporulation under the control of the transcription factor σ^E^, halts cytokinesis by inhibiting FtsZ (Handler et al., 2008). Finally, several phage-encoded polypeptides interact with FtsZ and delay the host cell division (Ballesteros-Plaza et al., 2013; Kiro et al., 2013; Haeusser et al., 2014).

New antibiotics are urgently needed to fight the widespread emergence of pathogens resistant to current therapeutic options, aggravated by the diminished antibiotic discovery pipeline (Payne, 2008; Boucher et al., 2009; Lewis, 2012; Lin et al., 2015). Inhibition of cell division in *B. subtilis* does not initially inhibit growth, but after several mass doubling periods it leads to a block in DNA replication followed by a complete cell growth arrest; the quiescent cells enter in a terminal cell-cycle state from which they cannot recover when shifted to permissive conditions (Arjes et al., 2014). These findings strongly support targeting the bacterial cell division machinery for the discovery of new antibacterial agents. Chemical inhibition of FtsZ by small molecules can in fact impair cell division and eventually cause bacterial death, an antibacterial mechanism of action to be clinically explored. The substituted difluorobenzamide PC190723 (Haydon et al., 2008; Czaplewski et al., 2009; Tan et al., 2012) and a few analogs (Stokes et al., 2013, 2014, Kaul et al., 2015, 2016; Lepak et al., 2015) have shown potent activity in animal models of infection, which validated FtsZ as a target for new antibacterials. A relatively large number of compounds have been reported to inhibit the function of FtsZ in bacterial cell division, to perturb purified FtsZ polymerization or its GTPase activity (Anderson et al., 2012; Schaffner-Barbero et al., 2012; den Blaauwen et al., 2014). Some among these inhibitors have demonstrated antibacterial activity, although in many other cases the specificity, FtsZ binding sites and bacterial phenotypic effects have not been clarified. FtsZ GTPase and polymerization screens have also given some conflicting results.

Specific cell-based screens other than simple filament formation are required to seek FtsZ-targeting inhibitors, but they are scarce (Stokes et al., 2005). Bacterial cytological profiling is a rapid and powerful approach for identifying the cellular mechanism of action of antibacterial molecules. It allows distinguishing antibiotics affecting different cellular pathways as well as different targets within the same pathway, using quantitative fluorescence microscopy, without the need for slow and labor-intensive analysis (Lamsa et al., 2012; Nonejuie et al., 2013). Cytological profiling has also been used for rapidly determining antibiotic susceptibility of clinical isolates of *Staphylococcus aureus* (Quach et al., 2016). In this work, we have characterized the effects on *B. subtilis* cells of a set of known FtsZ inhibitors and have defined their specific cytological profile; this should allow identifying new molecules targeting FtsZ in primary screening. Using this approach we have discovered that exogenously added synthetic peptide MciZ is an effective inhibitor of FtsZ localization and cell division in *B. subtilis*.

## Materials and Methods

### Strains and Fluorescence Microscopy

*Bacillus subtilis* 168 cells were grown in cation adjusted Mueller-Hinton broth (CAMHB; Becton, Dickinson and Company) at 37°C to an absorbance 0.1–0.2 at 600 nm and then the culture was divided into new flasks containing the compound at the desired concentration. After 3 h of incubation cells were directly observed or first stained with 4,6-diamino-2-phenylindole (DAPI, 0.25 μg/mL; Sigma) and FM4-64 (1 μg/mL; Sigma). For Z-ring analysis *B. subtilis* strain SU570 (Strauss et al., 2012), kindly donated by Dr. Elisabeth J. Harry (the ithree institute, University of Technology, Sydney, Australia), was grown in Antibiotic Medium 3 at 30°C, incubated with the compounds (1.5 h) and visualized after staining with DAPI and FM4-64.

*Escherichia coli* experiments were performed with the strain *envA1* (Young and Silver, 1991), provided by Merck Sharp & Dohme Corp. (Rahway, NJ, USA). For Z-ring visualization the arabinose inducible plasmid pFtsZ-YFP20 (chloramphenicol resistance) (Keffer et al., 2013), kindly donated by Dr. Carole A. Bewley (NIDDK, NIH, Bethesda, MD, USA), was transformed into strain *envA1* via electroporation, and colonies (termed envA1/pFtsZ-YFP) were selected after plating onto LB agar containing 20 μg/mL chloramphenicol. Replicate plates were made, and clones that expressed yellow fluorescence at mid-cell after 15 min induction with 0.4% L-arabinose in LB containing 20 μg/mL chloramphenicol were harvested and stored frozen as glycerol stocks at -80°C. FtsZ-YFP expression was induced as previously described (Keffer et al., 2013) and bacteria were incubated with the compounds (1.5 hour) at desired concentrations.

Aliquot of cells (5 μL) were harvested at appropriate time intervals and visualized with phase contrast or transferred to 1% (w/v) agarose pads, covered, and imaged with fluorescence, using a Zeiss Axioplan microscope equipped with 40x and 100× objectives and a Hamamatsu 4742-95 CCD camera. The fluorescent images in **Figures 4**, **6**, **7A** and **9** are contrast inverted for presentation purposes.

### FtsZ Inhibitors and Antibiotics

Small molecule FtsZ inhibitors (Supplementary Table S1) were synthesized by the Medicinal Chemistry Laboratory (Dr. María L. López-Rodríguez, Universidad Complutense de Madrid, UCM, Spain) as previously described (Andreu et al., 2010; Ruiz-Avila et al., 2013; Artola et al., 2015), except hemi-chrysophaentin (Keffer et al., 2013) that was kindly provided by Dr. C. Bewley (NIDDK-NIH, Bethesda, MD, USA) and zantrin Z3 that was acquired from the Mcule online drug discovery platform. These compounds were dissolved in dimethyl sulfoxide (DMSO) at appropriate stock concentrations. Residual DMSO in treated cultures and controls was less than 1% in all cases. Peptides MciZ and CRAMP, with the sequences MK VHRMPKGVVLVGKAWEIRAKLKEYGRTFQYVKDWISKP and ISRLAGLLRKGGEKIGEKLKKIGQKIKNFFQKLVPQPE, respectively, were synthesized in a Liberty Blue instrument (CEM Corp., Matthews, NC) using optimized, microwave-assisted solid-phase protocols and purified by HPLC to > 97% homogeneity. An independently synthesized MciZ batch from commercial sources (Peptide Specialty Laboratories GmbH, Heidelberg, Germany) was also used, of comparable purity and giving identical results in filamentation and Z-ring tests. For assays, peptides were dissolved in distilled water before use. MciZ concentration was measured spectrophotometrically employing an extinction coefficient 13980 M^-1^cm^-1^ at 280 nm, calculated from the amino acid sequence. Solutions of antibiotics were prepared in water or DMSO as recommended by the manufacturers. Nisin, vancomycin, carbonyl cyanide *m*-chlorophenyl hydrazone (CCCP), kanamycin, cerulenin, mitomycin C, cefotaxime and piperacillin (Sigma) were added to the growing bacterial cultures. In the case of daptomycin (provided by Novartis Pharma AG, Switzerland), extra calcium up to 50 μg/ml was employed for optimal activity.

FtsZ inhibitors were employed (unless otherwise indicated) at the minimal concentration that effectively induced filamentation while permitting initial *B. subtilis* growth (Artola et al., 2015): UCM62 5 μM, UCM78 4 μM, UCM79 4 μM, UCM81 3.5 μM, UCM82 25 μM, UCM93 7.5 μM, UCM95 12.5 μM. PC170942 was employed at 20 μM (Ruiz-Avila et al., 2013); hemi-chrysophaentin (Keffer et al., 2013) at 18 μM, PC190723 at 5.6 μM (Haydon et al., 2008) and MciZ at 1 μM (tested from 0.5 to 40 μM in this work). *B. subtilis* mass doubling period values were determined from least squares fits of Log (Absorbance 600 nm) versus time in the initial linear region. The fragments of PC190723 (CTPM and DFMBA Andreu et al., 2010), were employed at 1mM concentration. Concentrations of other compounds were: CRAMP 5 μM, zantrin Z3 3.5 μM, totarol 10 μM. Concentrations of antibiotics nisin (10 mg/L), CCCP (100 μM), vancomycin (250 μg/L), and daptomycin (1 mg/L) were those previously used in *B. subtilis* cytological studies (Lamsa et al., 2012; Pogliano et al., 2012). Concentrations of kanamycin (10 mg/L), cerulenin (15 mg/L), cefotaxime (0.75 mg/L), piperacillin (2 mg/L), and mitomycin C (0.1 mg/L) were adapted from those used in *E. coli* cytological profiling (Nonejuie et al., 2013). After 3 hours of incubation with antibiotics increased optical density of the cultures was observed, except in the treatments with CCCP, kanamycin and daptomycin, for which cell lengths were measured in the remaining not lysed cells.

### Membrane Integrity of *B. subtilis* Cells

The effect of FtsZ inhibitors on membrane integrity was monitored using the Live/Dead BacLight bacterial viability kit (Molecular Probes). *B. subtilis* 168 cells were grown at 37°C until absorbance values of 0.2-0.3 and then grown in the absence and presence of FtsZ inhibitors for an additional 30 min. Then, cells were pelleted down, washed with 0.85% NaCl solution and incubated with 18 μM propidium iodide for 30 min at 25°C in 0.85 % NaCl solution. After an additional wash cell suspensions were excited at 470 nm and fluorescence spectra were recorded in the range of 500-700 nm.

### Membrane Potential of *B. subtilis* Cells

*Bacillus subtilis* 168 cells were grown in Mueller-Hinton supplemented with 0.2% glucose to absorbance 0.1, incubated in the absence or presence of FtsZ inhibitors for 15 minutes and then with 30 μM of 3,3′-diethyloxa-carbocyanine iodide (DiOC_2_, BacLight Bacterial Membrane Potential Kit from Molecular Probes) for 30 min at 25°C. After two washes with phosphate buffered saline pH 7.4 supplemented with 0.1% glucose, fluorescence emission was measured using 488 nm as the excitation wavelength. The ratio of red (575 nm) to green (530 nm) fluorescence intensity was used as an indicator of membrane potential.

### Cytological Profiling

Cell parameters were measured using the analysis tools of Wasabi software (Hamamatsu). Line profiles were drawn on cell stained with FM4-64 to measure cell length distinguishing from potential cell chaining. DNA staining with DAPI was employed to measure nucleoid length. For Z-rings cytological analysis, we operatively defined a Z-ring as a continuous band of FtsZ-GFP across the width of the cell; the distance between Z-rings was measured using the line profile analysis tool. We defined FtsZ *foci* as any (punctate or large) accumulation of FtsZ-GFP that does not fulfill the criteria for a Z-ring.

Cell parameters for each treatment were obtained from three or more independent experiments. Before statistical Student’s *t*-test, outliers were removed (Lower limit = Q1-1.5(IQR); Upper limit = Q3 + 1.5(IQR). The deviation represented in graphics corresponds to the standard error. Principal component analysis (PCA) and hierarchical cluster analysis were performed using the XLSTAT (version 2015.1.02) program on Microsoft Excel for Windows.

### Biochemical Methods

*Bacillus subtilis* FtsZ and *E. coli* FtsZ proteins were purified, and their polymerization characterized by sedimentation, light scattering and GTPase activity assays in 50 mM Hepes/KOH, 50 mM KCl, 1 mM EDTA, 10 mM MgCl_2_, 1 mM GTP, pH 6.8 at 25 °C as previously described (Ruiz-Avila et al., 2013).

## Results and Discussion

**FIGURE 1:**
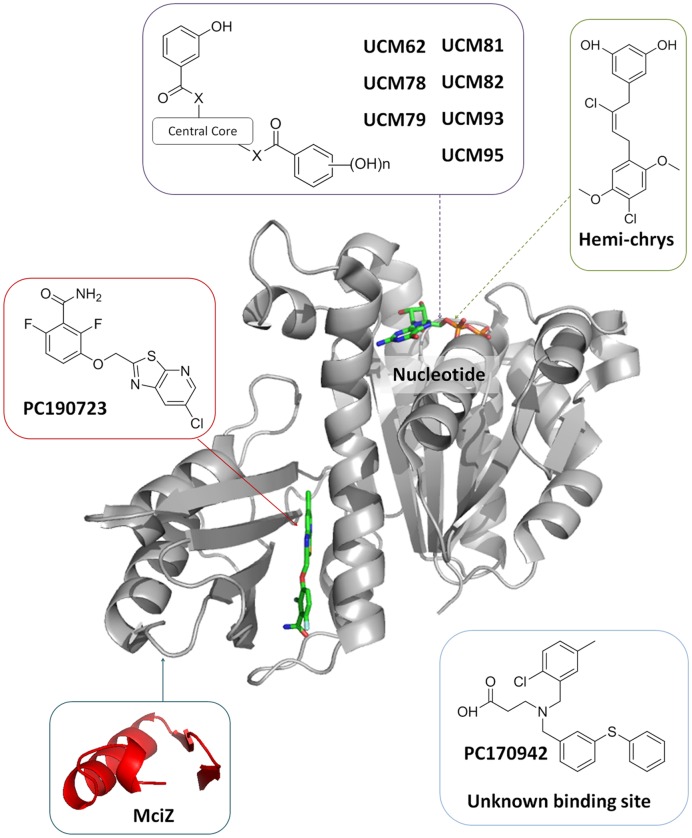
**FtsZ inhibitors used in the cytological profile analysis.** Schematic representation of inhibitors binding sites on FtsZ from *Bacillus subtilis* (based on Protein Data Bank entries 4DXD and 4U39). Notice that PC190723 requires the binding cleft to open (Elsen et al., 2012) See full compound list and antibacterial activities in Supplementary Table S1. Peptide MciZ (Bisson-Filho et al., 2015) was included in this scheme after the activity of synthetic MciZ on cell division was found (See Results).

To define the cytological profile of chemical FtsZ inhibitors we selected molecules that bind to different sites in FtsZ (**Figure 1**): (i) GTP-replacing FtsZ inhibitors from our in-house synthetic library (UCM62, UCM78, UCM79, UCM81, UCM82, UCM93, UCM95) (Artola et al., 2015); (ii) a GTP-replacing fragment of the natural FtsZ inhibitor crysophaentin (Hemi-chrys; Keffer et al., 2013); (iii) the well-known inhibitor PC190723 that binds in the cleft between FtsZ’s nucleotide binding and C-terminal domains (Haydon et al., 2008; Andreu et al., 2010; Adams et al., 2011; Elsen et al., 2012), and (iv) the effective inhibitor PC170942 (Stokes et al., 2005) binding to an undetermined site on FtsZ (Ruiz-Avila et al., 2013). Each of the small molecules in classes (i) to (iv) has been documented to possess antibacterial activity on *B. subtilis* and methycilin resistant *S. aureus* (MRSA) (Supplementary Table S1).

### Defining a Relevant Filamentous Phenotype for FtsZ Inhibitors in *B. subtilis*

**FIGURE 2:**
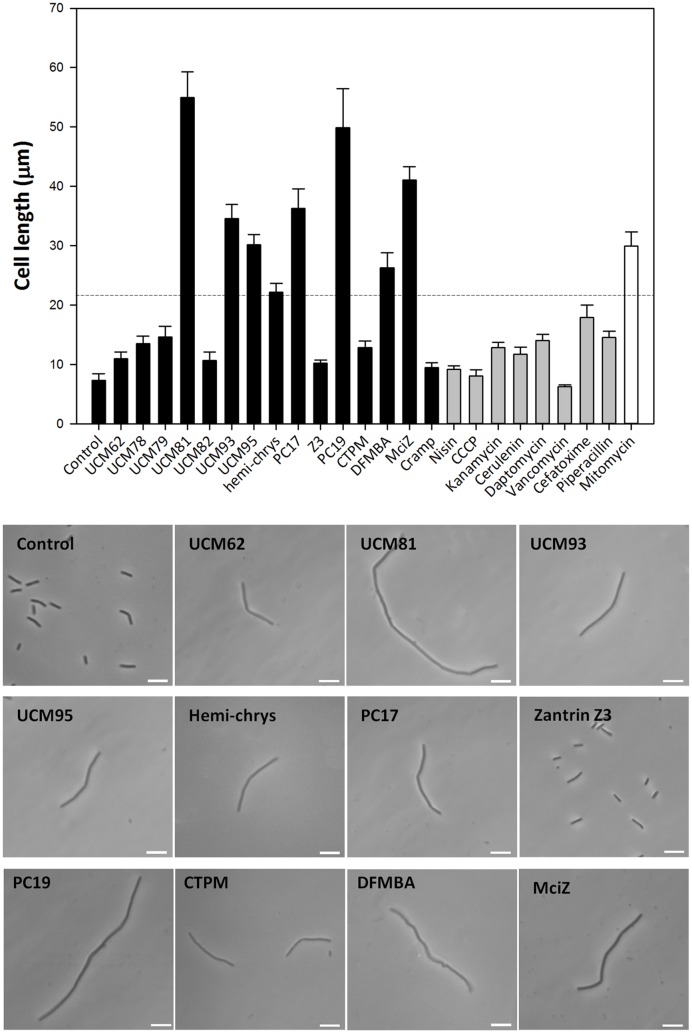
**Effect of FtsZ inhibitors and antibiotics with different mode of action in cell division.** Cells of *B. subtilis* 168 were incubated for 3 h with the compounds, each one at its effective concentration, and cell length was measured (see Materials and Methods). Significant increases compared to the treatments with control antibiotics -with the exception of mitomycin- were found with compounds UCM81, UCM93, UCM95, hemi-chrysophaentin, PC170942 (PC17), MciZ, PC190723 (PC19), and DFMBA (*p* < 0.05; dotted line). The histogram shows average and standard error from three independent experiments with more than 30 measurements each. Representative examples of undivided individual cells observed upon treatment with the compounds are shown in phase contrast. Scale bar: 10 μm.

One hallmark of bacterial cell division inhibition is the induction of an enlarged phenotype, caused by the initially continuing growth without division. In order to quantitatively characterize this effect we measured the length of wild type *B. subtilis* 168 cells incubated with our FtsZ inhibitor panel, as well as with several known antibiotics of different mechanisms of action. The treatment with each of the above FtsZ inhibitors produced a statistically significant increase of the average cell length with respect to the untreated control (**Figure 2**, black bars), at effective concentrations not markedly affecting initial growth (except UCM95 and PC170942; Supplementary Table S1). However, several of the antibiotics that do not act on cell division but target cell wall (vancomycin, cefotaxime, and piperacillin), cell membrane (cerulenin and daptomycin) or protein synthesis (kanamycin) also produced significantly elongated cells (**Figure 2**, gray bars). For that, a more stringent criterion to identify relevant FtsZ inhibitors is an increase of the cell length significantly larger than the non specific increase produced by antibiotics. This is shown by the dash line in **Figure 2**, which corresponds approximately to three times the length of the not treated *B. subtilis* cells. On the other hand, DNA cross-linker antibiotics such as mitomycin C, indirectly inhibit cell division leading to a marked increase in cell length (**Figure 2**, white bar). However, criteria such as nucleoid length or Z-ring morphology, allow distinguishing them from FtsZ inhibitors as will be shown later. Notice that other growth conditions or bacterial strains may give different results, but we found always advisable the comparison with control antibiotics not acting on cell division. In our case, the small molecule inhibitors that fulfill the set requirement with probability *p* < 0.05 are: UCM81, UCM93, UCM95, hemi-chrysophaentin, PC170942, DFMBA, and PC190723.

We also tested other FtsZ inhibitors from the literature, such as zantrin Z3 (Margalit et al., 2004; Anderson et al., 2012) but a lack of effect on *B. subtilis* cell length was observed. During these experiments, we unexpectedly found that the addition of synthetic peptide MciZ (1-5 μM) to the growing medium induces a marked filamentation of *B. subtilis*. Previous studies had shown that intracellular MciZ expression leads to the formation of filaments that are deficient in Z-rings (Handler et al., 2008; Bisson-Filho et al., 2015), but the effects of exogenous MciZ had not been observed. We also analyzed the effect of the antimicrobial peptide CRAMP, which shares part of the MciZ sequence, but found an insignificant increase in average cell length (9.4 ± 0.8 μm) with respect to the control (7.3 ± 1.1 μm; *p* > 0.5), quite below the threshold (17.9 ± 2.1 μm) of relevant filamentation of *B. subtili*s cells (**Figure 2**), in agreement with previous results (Handler et al., 2008). For convenience, the cytological effects of MciZ are shown below with those of the small molecule inhibitors, but they will be separately analyzed and discussed later.

### The Biochemical Profiles of PC190723 Fragments Explain Their Cellular Activities

**FIGURE 3:**
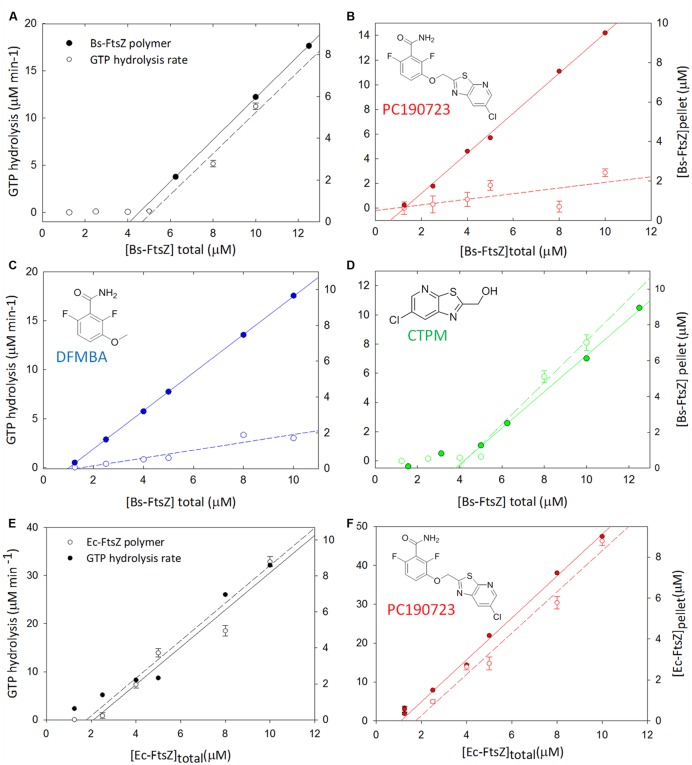
**Effect of PC190723 and its fragments on assembly and GTPase activity of BsFtsZ and EcFtsZ. (A-D)** Graphs showing the relationship between the sedimentation of BsFtsZ polymers and the rate of hydrolysis in the presence of GTP (2 mM) and no compound **(A)** or presence of PC190723 15 μM **(B)**, DFMBA 4 mM **(C)**, and CTMP 1 mM **(D)**. E-F experiments with EcFtsZ showing the correlation of GTPase activity with polymer concentration in the presence of GTP (2 mM) and the absence of compound **(E)** or presence of 15 μM PC190723 **(F)**. For Cr and GTPase activity values see Supplementary Table S2.

The PC190723 fragment DFMBA (2,6-difluoro-3-hydroxybenzamide) was included in the group of active compounds inducing *B. subtilis* cell filamentation. DFMBA appears responsible for the activity of PC190723, as the other moiety CTPM ((6-chloro [1,3]tiazol[5,4-b]pyridin-2-yl) methanol), did not fulfill the *p* < 0.05 criterion for relevant cell elongation (**Figure 2**). In order to explain these cellular results and to improve biochemical profiling methods for FtsZ inhibitors, we have analyzed in detail the effects of PC190723 and its fragments on the polymerization and GTPase activity of FtsZ from *B. subtilis*. GTPase activity is a consequence of FtsZ assembly, because the active site is completed between consecutive monomers in FtsZ filaments. We have thus observed a direct correlation between the bulk GTPase rate and BsFtsZ polymer concentration in the solution (**Figure 3A**). Both appear above a critical BsFtsZ concentration (Cr, the minimal concentration of protein monomers needed for cooperative polymerization) and then grow linearly with the total protein concentration. This parallelism is abrogated by PC190723, due to an inhibition of the GTPase activity of BsFtsZ polymers and the decrease in Cr by polymer stabilization (**Figure 3B**; Supplementary Table S2). The PC190723 fragment DFMBA reduced Cr with respect to control and inhibited the GTPase activity (**Figure 3C**; Supplementary Table S2), similarly to PC190723. However, the fragment CTPM only weakly modified GTPase activity and Cr (**Figure 3D**; Supplementary Table S2). These results indicate that DFMBA is the active moiety of PC190723, whereas the CTPM part enhances binding (Andreu et al., 2010) by means of hydrophobic interactions (Tan et al., 2012), providing an explanation for the cell filamentation results.

Notice that conflicting results were reported (Anderson et al., 2012) when attempting to reproduce previously observed FtsZ GTPase activity changes induced by PC190723 (Haydon et al., 2008; Andreu et al., 2010). This may be explained by the results in **Figure 3** that show how apparent GTPase activation, inhibition or weak effects relative to controls can be measured if single FtsZ concentrations are employed. Simplified biochemical tests with FtsZ assembly modulators may give complicated results, lead to unproductive screens or to conflicting interpretations, particularly for individual GTPase activity assays in the absence of other information. When a suitable binding assay is available (Ruiz-Avila et al., 2013), we prefer determination of specific binding affinity to FtsZ, which in fact predicted antibacterial activity in the UCM inhibitor series (Artola et al., 2015).

### FtsZ-Targeting Cell Division Inhibitors Induce Aberrantly Positioned Z-Rings

**FIGURE 4:**
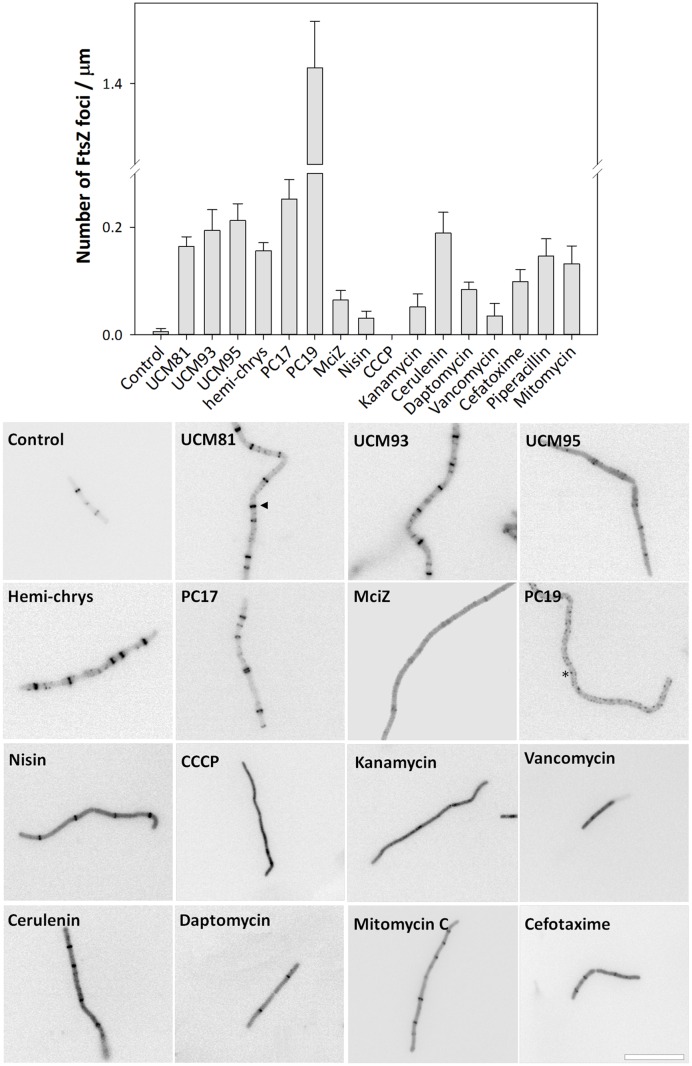
**Effects of FtsZ inhibitors on FtsZ subcellular localization.** Cells of *B. subtilis* SU570 (FtsZ-GFP) were incubated for 1.5 h with the different compounds and visualized under the fluorescence microscope. Z-rings and FtsZ *foci* were observed (see Materials and Methods, the triangle and the asterisk indicate examples of ring and *foci*, respectively). The number of *foci* per micron were quantified (graphic; average and standard error are shown, *n* = 3). Scale bar: 10 μm.

The effects of the selected inhibitors in FtsZ subcellular localization was analyzed in *B. subtilis* SU570, a strain that has FtsZ fused to green fluorescent protein (FtsZ-GFP) as the only FtsZ protein (Strauss et al., 2012). The GTP-replacing inhibitors, as well as PC170942, produced alterations in the regular midcell distribution of Z-rings and the appearance of punctate FtsZ *foci* (**Figure 4**; see Materials and Methods). PC190723 produced a large number of *foci* throughout the cells lacking Z-rings, in agreement with the known effects of benzamide inhibitors (Haydon et al., 2008; Adams et al., 2011). However, hemi-chrysophaentin and PC170942 increased the number of Z-rings, and MciZ decreased it, whereas no significant difference was found with the other inhibitors (Supplementary Table S3). Cells exposed to zantrin Z3 showed similar Z-rings to control cells. With the aim to verify if the observed phenotypes were specific for FtsZ inhibitors, *B. subtilis* SU570 cells were exposed to different antibiotics. CCCP, kanamycin and vancomycin altered the distribution of the Z-rings; nisin, daptomycin, cefotaxime, and mitomycin C did not, and cerulenin caused a marked appearance of FtsZ *foci*. Quantification of the number of *foci* (**Figure 4**) showed that, except in the case of inhibitors PC190723 and PC170942, the number of FtsZ *foci* was significantly greater than in the control but no larger than observed with cerulenin. Therefore, FtsZ *foci* should not be considered as a single adequate criterion to differentiate specific FtsZ inhibitors.

**FIGURE 5:**
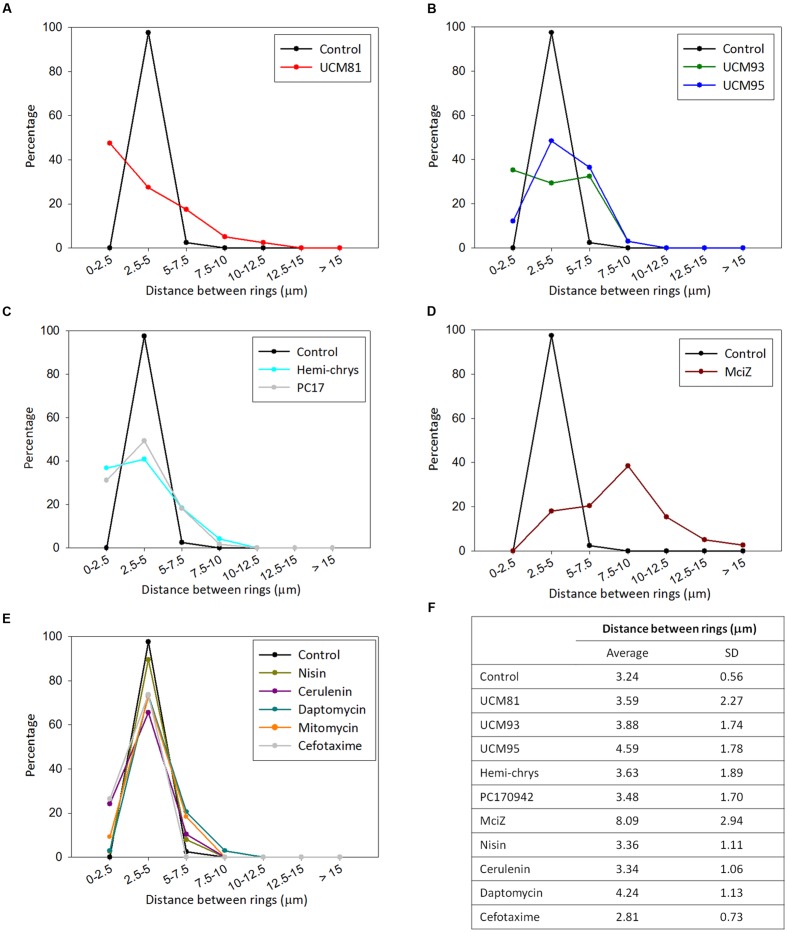
**Distribution of Z-rings along the cell. (A-E)** The distance between pairs of consecutive rings in cells of *B. subtilis* SU570 exposed to the compounds were measured, classified in seven groups of distance (from 0 to 2.5 μm, from 2.5 to 5 μm, from 5 to 7.5 μm, from 7.5 to 10 μm, from 10 to 12.5 μm, from 12.5 to 15 μm, and more than 15 μm) and the percentage of rings in each group were represented. **(F)** The average and standard deviation of the distance between rings are shown in the table to point out the spread of the distribution.

We found that analyzing the distribution of rings throughout the cell allowed to clearly differentiate cells treated with FtsZ inhibitors from untreated controls and from cells treated with other antibiotics (**Figure 5**). Control cells had regularly distributed Z-rings and most of the rings (97%) were normally spaced at distances comprised between 2.5 and 5 μm. Treatment with FtsZ inhibitors induced wider, aberrant Z-ring distributions, reducing the proportion of normally spaced rings and increasing the percentage of closer or more distant rings, particularly in cells treated with UCM81 (**Figure 5A**) or with MciZ (**Figure 5D**). The other inhibitors were classified as producing intermediate effects (**Figures 5B,C**). Finally, in cells treated with control antibiotics (nisin, cerulenin, daptomycin, cefotaxime and mitomycin C) more than 60% of the rings were separated a distance of 2.5-5 μm similarly to the untreated control (**Figure 5E**). Therefore, we conclude that specific FtsZ inhibitors are characterized by either suppressing ring formation or causing an aberrant distribution of rings along the cell, consisting of an increase of closer rings or more distant rings at the expense of the correctly positioned mid-cell rings.

### Specific FtsZ Inhibitors Induce Membrane Spots Without Affecting Membrane Potential and Permeability

**FIGURE 6:**
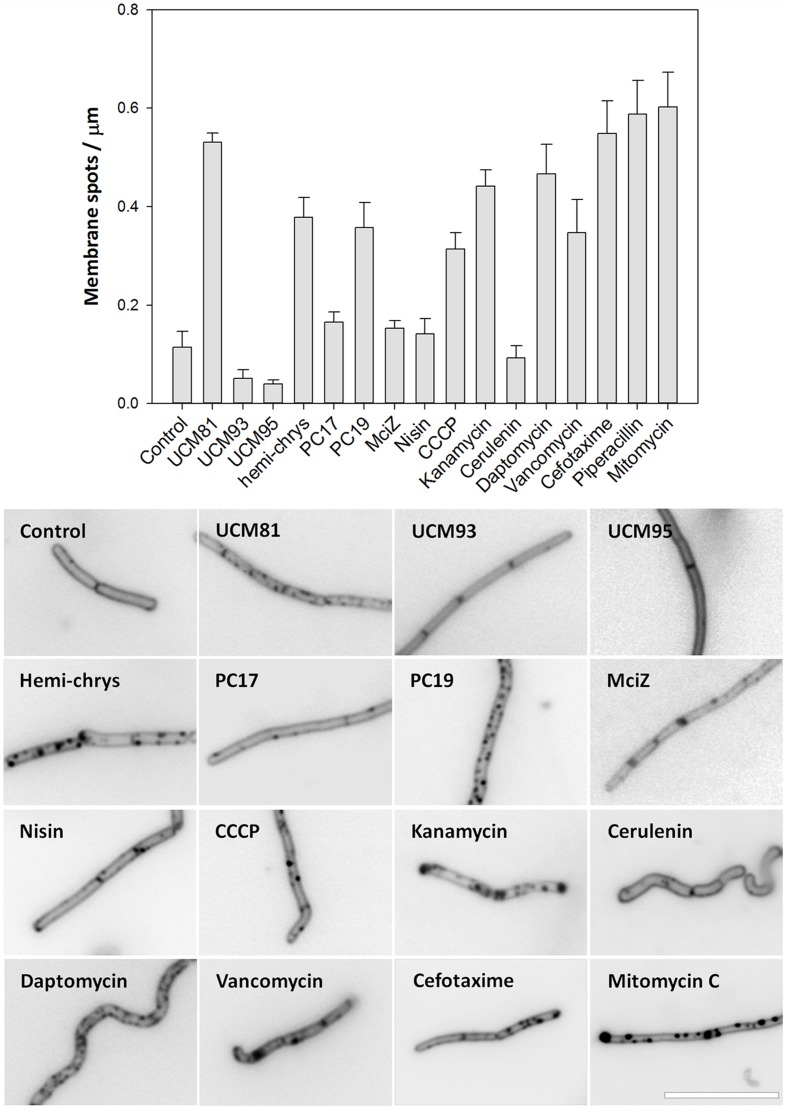
**Membrane morphology in cells treated with FtsZ inhibitors and other antibiotics.** *B. subtilis* 168 cells grown in the presence or absence of compounds during 3 h and before microscopic analysis they were stained with 5 μg/mL of FM4-64. The number of small membrane spots per micron were determined (graphic; average and standard error are shown, *n* = 3). Scale bar: 10 μm.

Next steps on the cytological characterization of FtsZ inhibitors were to determine their possible effect on membrane morphology and membrane permeability. To visualize membranes we used the vital stain FM4-64. As can be observed in **Figure 6**, inhibitors UCM81, hemi-chrysophaentin and PC190723 caused a significant increase of membrane stained spots randomly distributed through the cell. By contrast, the membrane morphology observed in cells treated with inhibitors UCM93, UCM95, and PC170942 was more similar to control cells. MciZ frequently increased membrane staining around division septa rather than inducing disperse membrane spots. Following the trend of our study we also analyzed the effect of different known antibiotics on membrane morphology. Vancomycin, CCCP, kanamycin, and daptomycin induced abundant membrane spots compatible with previously observed morphologies (Lamsa et al., 2012; Pogliano et al., 2012; Nonejuie et al., 2013). Thus, membrane spots by themselves do not allow discerning specific FtsZ inhibitors from the non-specific effects of other antibiotics.

To test whether FtsZ inhibitors increased the permeability of *B. subtilis* membranes we used propidium iodide (PI) (Supplementary Figure S1A). The cellular uptake of PI increases with increasing membrane permeability and then this dye emits red fluorescence (γ_emission_ = 620 nm) upon binding to DNA. When *B. subtilis* cells were grown with FtsZ inhibitors and stained with PI the red fluorescence was similar to control cells. When cells were treated with nisin (10 mg/L) the red fluorescence detected was significantly higher than in the control. Protonophores such as CCCP alter membrane potential, but do not allow passage of other solutes. Thus, PI fluorescence did not increase in cells treated with CCCP (Supplementary Figure S1A). This response is similar to antibiotics such as daptomycin, which increases the flux of K+ ions across the membrane, but has not effect on PI uptake (Silverman et al., 2003). Alkyl gallate FtsZ inhibitors affect membrane integrity (Krol et al., 2015), however, our effective FtsZ inhibitors do not perturb membrane integrity of *B. subtilis* cells.

It has been noted that disruption of membrane potential inhibits cell division by detachment of the divisomal machinery from the membrane (Strahl and Hamoen, 2010) and several FtsZ inhibitors have been reported to alter membrane potential and permeability (Foss et al., 2013). To determine if our selected FtsZ inhibitors altered the membrane potential, cells of *B. subtilis* 168 were incubated with the compounds and stained with 3,3′-diethyloxa-carbocyanine iodide (DiOC_2_), a fluorophore that emits green fluorescence in solution and shifts toward the red when concentrated at the cell membrane. Values of red to green fluorescence emission ratio are indicative of the membrane potential. The fluorescence ratio I_575_/I_530_ in cells treated with FtsZ inhibitors was similar to control cells, except for decreases observed with PC170942 and with totarol, a compound that alters membrane potential and permeability and thus affects FtsZ localization (Foss et al., 2013). CCCP (10 μM), a known membrane potential inhibitor, decreased I_575_/I_530_ significantly (*p* < 0.01) compared to the control (Supplementary Figure S1B).

### FtsZ Inhibitors Modify Nucleoid Morphology

**FIGURE 7:**
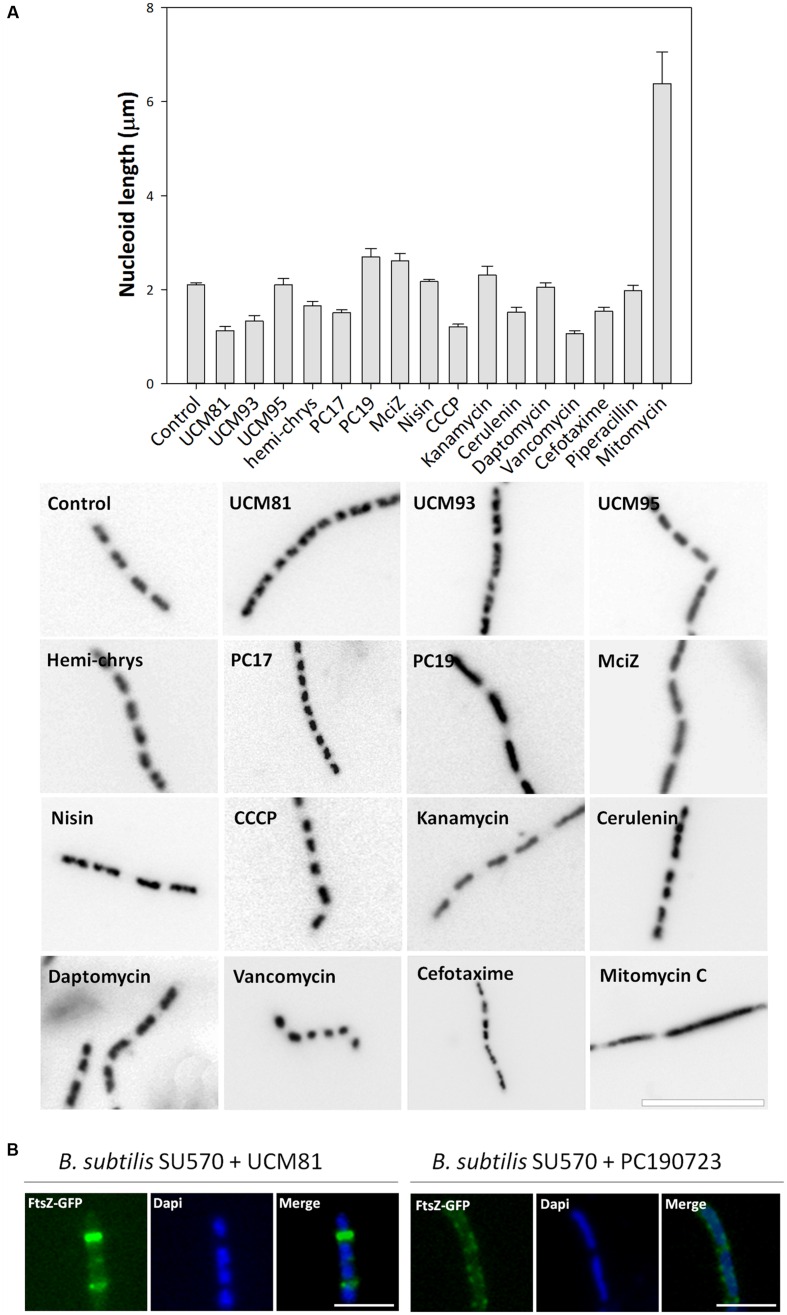
**Nucleoid morphology in cells treated with FtsZ inhibitors and others antibiotics. (A)** *B. subtilis* 168 cells grown during 3 h in the presence or absence of compounds were stained with DAPI (1 μg/mL) and analyzed under the microscope. Nucleoid length was measured (graphic; average and standard error are shown, n = 3). Scale bar: 10 μm. **(B)** Detailed views of FtsZ-GFP and nucleoid morphology in *B. subtilis* SU570 cells grown during 1.5 hours in the presence of UCM81 or PC190723. Scale bar: 4 μm.

To analyze nucleoid morphology, *B. subtilis* 168 cells treated with the selected inhibitors were stained with DAPI and visualized under the microscope. Two different effects of the FtsZ inhibitors were observed (**Figure 7A**): UCM81, UCM93, hemi-chrysophaentin, and PC170942 lead to fragmented short nucleoids compared to control cells; by contrast, the treatments with PC190723 or MciZ provoked the appearance of longer nucleoids than in control cells. Quantitative analysis of these observations, determining nucleoid length, supported the existence of these two distinct groups of inhibitors (**Figure 7A**, graphic). The more marked nucleoid fragmentation was caused by UCM81, which in turn was the inhibitor for which a larger number of closer rings were detected (**Figure 5A**). In this case, abnormally close FtsZ-GFP rings match nucleoid constriction zones; and nucleoid constrictions where rings are not observed may correspond to aborted rings, or to previous rings that have already disappeared (**Figure 7B**). On the other hand, longer nucleoids correlated with more spaced rings with MciZ, or with absence of rings with PC190723. The enlarged nucleoids with PC190723 extend among non-constricting FtsZ-GFP *foci* (**Figure 7B**). Thus, each class of abnormal nucleoid morphology might reflect a different impairment of the cell division ring: constricted nucleoids may be caused by abnormally close Z-rings whereas the elongated nucleoids might be suggested to fill the regions without Z-rings. Alternatively, the effects on the Z-ring could be attributed to off-target changes in nucleoid shape (via nucleoid occlusion activity); however, this appears quite unlikely for the best characterized FtsZ inhibitor PC190723, the regulator MciZ and the chemically different UCM81 and PC170942 inhibitors all together. In practice, using nucleoid fragmentation alone we could not distinguish FtsZ inhibition from similar effects caused by three control antibiotics (CCCP, cerulenin, vancomycin; **Figure 7A**), however, the latter could be distinguished by the lack of significant filamentation (**Figure 2**). On the other hand, the very long nucleoids observed with mitomycin C distinguish this DNA targeting antibiotic from FtsZ inhibitors (**Figure 7A**), although both induced comparable filamentous phenotypes (**Figure 2**).

### A Lack of Effective Inhibitors of *E. coli* Cell Division Targeting FtsZ

The cytological effects of FtsZ inhibitors were also characterized in the Gram-negative bacterium *E. coli*. To overcome the physical barrier imposed by the outer membrane we used the *envA1 E. coli* strain that is permeable to antibiotics and dyes (Young and Silver, 1991). Only with compounds UCM81 (5 μM), PC190723 (10 μM), CRAMP (10 μM), and hemi-chrysophaentin (340 μM) we found some undivided longer cells (Supplementary Figure S2), representing only 3-5% of the total cell population. These minority long cells showed extensively fragmented nucleoids, altered Z-ring distributions and some FtsZ *foci* (Supplementary Figure S2). The Z-ring impairment observed with hemi-chrysophaentin was similar to the previously reported morphology (Keffer et al., 2013). These effects appeared specific of FtsZ inhibitors because treatment with control antibiotics did not affect Z-rings (Supplementary Figure S3). We report these effects on a minority of cells solely as examples of practically negative results that could be confused with a relevant FtsZ inhibition if the whole cell population was not analyzed.

It has been reported that TXY436, a prodrug of PC190723, induces changes in the morphology of *E. coli* and *Klebsiella pneumoniae* consistent with inhibition of cell division when the RND-type efflux pump activity is genetically or chemically inhibited (Kaul et al., 2014). Consequently, we evaluated the possible effect of our selected FtsZ inhibitors in the *E. coli* MG1622 wild type strain cultured in the presence of the efflux pump inhibitor PAβN (Pages et al., 2005). However, the results obtained were similar to those with the *envA1* strain, in which no significant effects of the FtsZ inhibitor on average cell length were observed.

We further investigated the causes for the lack of susceptibility of *E. coli* to PC190723, analyzing its effects on GTPase activity and polymerization of FtsZ from *E. coli* (EcFtsZ) (**Figure 3E**). In this case, PC190723 did not disable the correlation between GTPase activity and polymerization as for BsFtsZ (compare **Figure 3F** with **Figure 3B**), because it insignificantly modified Cr and only weakly increased the GTPase activity of EcFtsZ polymers (Supplementary Table S2). The small GTPase increase has been attributed to non-specific or ineffective binding of PC190723 to EcFtsZ (Andreu et al., 2010). The biochemical profiles of PC190723 on BsFtsZ and EcFtsZ thus explain the irrelevant effect that we have found on the *envA1 E. coli* permeable cells. They support the susceptibility of certain Gram-positive bacteria compared to the resistance of Gram-negative bacteria to PC190723, which tracks to having Val307 at the ligand binding site rather than Arg or His residues (Haydon et al., 2008).

In conclusion, although we could document cytological alterations induced by selected FtsZ inhibitors on *E. coli* cells, the affected cells are only a small fraction not representative of the population, rendering the compounds essentially ineffective in this Gram-negative bacterium. Our cytological and biochemical results with selected inhibitors underscore a current scarcity of effective FtsZ-targeting inhibitors for Gram-negative bacteria, although we cannot exclude that other compounds from the literature may work.

### Categorizing FtsZ Inhibitors and Synthetic Peptide MciZ in *B. subtilis*

**FIGURE 8:**
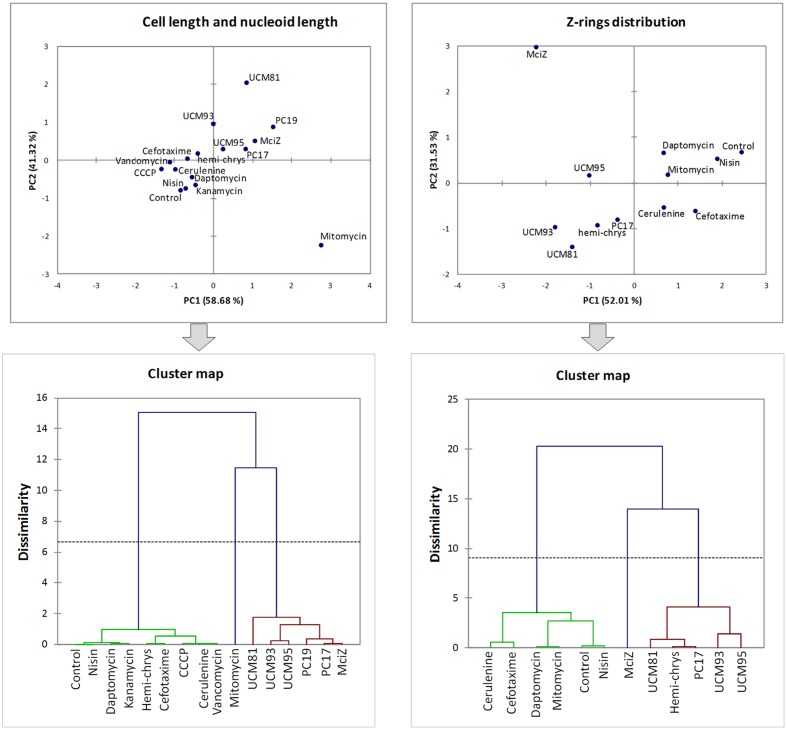
**Principal component analysis (PCA) results.** PCA graphs showing PC1 and PC2, using unweighted variables cell length and nucleoid length (left graph) or Z-rings distribution (right graph). Lower graphs correspond to dendrograms obtained after a hierarchical cluster analysis based on PC values from the PCAs.

To quantitatively analyze the set of results obtained with *B. subtilis* cells we performed principal component analysis (PCA) based on our cytological parameters: cell length, number of Z-rings/μm, number of FtsZ *foci*/μm, number of membrane spots/μm, nucleoid length and Z-rings distance distribution in four intervals (Supplementary Table S4). The results obtained from this analysis allowed us to classify cells with similar morphologies and establish which variables are necessary and sufficient to differentiate specific FtsZ inhibitors from non-specific antibiotic effects, as well as FtsZ inhibitors among them. As was observed previously in the individual analysis of the number of membrane spots or FtsZ *foci*, including these variables in the PCA did not make possible to differentiate FtsZ inhibitors from other antibiotics. Then, removing variables in successive PCA tests lead to determine a minimal set of variables that are enough to categorize FtsZ inhibitors (**Figure 8**). PCA based on variables “cell length” and “nucleoid length” allows to distinguish FtsZ inhibitors from other antibiotics, with the exception of hemi-chrysophaentin (the less effective FtsZ inhibitor included). Using the variable “Z-ring distribution” in the PCA lead to a complete separation of FtsZ inhibitors form other antibiotics, although this has the disadvantage that it is not applicable to those inhibitors that cause the complete absence of Z-rings such as PC190723, and it requires using a modified strain expressing FtsZ-FP or to carry out immunofluorescence assays.

The set of results obtained analyzing the cytological profile of FtsZ inhibitors compared with the phenotypic effects induced by other antibiotics allow to establish guidelines for identifying FtsZ inhibitors. There are two morphological parameters that should be analyzed for screening potential FtsZ inhibitors: the cell length and the nucleoid length. For further studies and categorizing FtsZ inhibitors identified in the screening we suggest analyzing the distribution of Z-rings.

**FIGURE 9:**
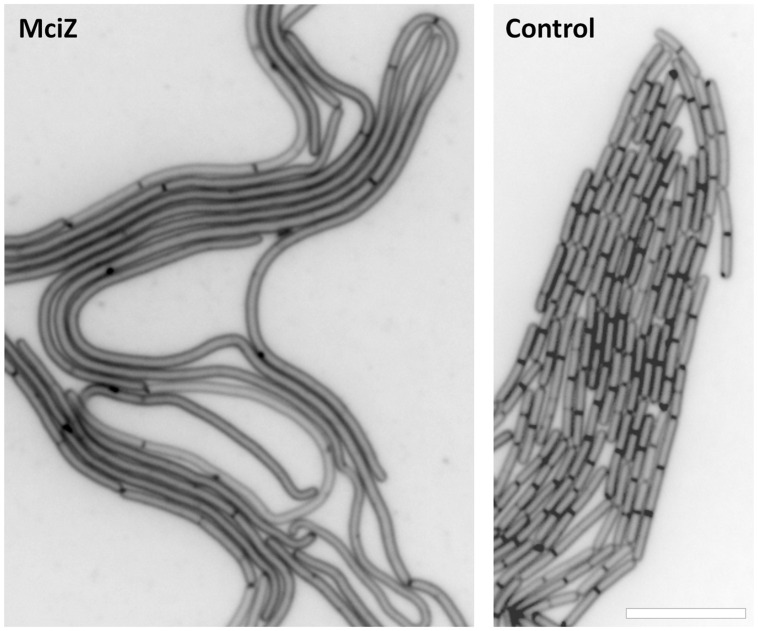
**Effect of synthetic MciZ on *B. subtilis* cell division.** Cells were grown for two hours on Muller-Hinton broth agar medium in the presence or absence of 2.5 μM MciZ in a Gene Frame system (Thermo Scientific). Membranes were visualized with FM4-64. Scale bar: 10 μm.

Using our cytological profile approach for FtsZ inhibitors we have identified exogenous MciZ as an effective inhibitor of *B. subtilis* cell division. The crystal structure of the FtsZ-MciZ complex has shown that MciZ blocks the C-terminal association interface of FtsZ, and a minus-end FtsZ filament capping mechanism has been proposed for this developmental regulator, which substoichiometrically inhibits FtsZ assembly when expressed in *B. subtilis* cells (Bisson-Filho et al., 2015). We have now observed that, similarly to the effects of several small molecule FtsZ inhibitors, MciZ addition to the culture medium leads to the formation of long undivided cells (**Figure 2**), but these have quite sparsely distributed Z-rings along them (**Figures 4** and **5D**). MciZ did not induce membrane spots (**Figure 6**) and did not impair membrane potential (Supplementary Figure S1B). MciZ increased the nucleoid length (**Figure 7**), reflecting a longer distance between Z-rings than in the case of small molecule inhibitors, which categorized peptide MciZ as a separate class among FtsZ inhibitors (**Figure 8**). This new finding validates our cytological profiling approach for FtsZ inhibitors. The remarkable filamentation effect of exogenous MciZ on *B. subtilis* (**Figure 9**), resembling the endogenous MciZ effects (Handler et al., 2008; Bisson-Filho et al., 2015), raises the question of how MciZ penetrates the cells and suggests new possibilities for designing synthetic peptide inhibitors of bacterial cell division.

## Conclusion

We have determined the cytological effects caused by selected cell division inhibitors targeting different binding sites of *B. subtili*s FtsZ. The analysis of cell length, Z-rings, nucleoid morphology, membrane morphology, and permeability allowed us to establish the cytological profile of chemical FtsZ inhibitors, as well as the criteria that distinguish them from antibiotics with other mechanisms of action. Quantifying cell length and nucleoid length should be sufficient to screen potential FtsZ inhibitors. The distribution of Z-rings may be employed for detailed studies and categorizing FtsZ inhibitors. In addition, biochemical profiling with FtsZ polymerization and GTPase assays may be used, as exemplified for PC190723 action on *B. subtilis* FtsZ and its lack of effect on the Gram-negative *E. coli* FtsZ; when possible, specific binding assays to determine the affinity of inhibitor binding to FtsZ are preferable. We have applied cytological profiling to the mother cell inhibitor MciZ, normally an endogenous regulator for sporulation, with the finding that exogenously added synthetic MciZ peptide is able to effectively inhibit cell division in *B. subtilis* cells by targeting FtsZ.

## Author Contributions

LA-B, DA, JA designed the work; LA-B, LR-A, SH performed experiments; LA-B, DA, SH, JA analyzed results; LA-B, JA wrote the paper with input from all authors, who revised and approved the final paper.

## Conflict of Interest Statement

The authors declare that the research was conducted in the absence of any commercial or financial relationships that could be construed as a potential conflict of interest.
