# Supplementary material for: Cytological Profile of Antibacterial FtsZ Inhibitors and Synthetic Peptide MciZ
Source: Front Microbiol. 2016 Oct 3;7:1558. doi: 10.3389/fmicb.2016.01558 (PMC5045927; doi:10.3389/fmicb.2016.01558)
Supplement: Supplementary file 1 [file Presentation_1.PDF]

Supplementary Material

Cytological profile of antibacterial FtsZ inhibitors and synthetic peptide MciZ

Lidia Araújo-Bazán[1]*, Laura B. Ruiz-Avila[1†], David Andreu[2], Sonia Huecas[1], José M. Andreu[1]*

[1] Centro de Investigaciones Biológicas, CSIC, Madrid, Spain
[2]Dept. of Experimental and Health Sciences, Universitat Pompeu Fabra, Barcelona, Spain

*** Correspondence:**
Lidia Araújo-Bazán, José M. Andreu
Centro de Investigaciones Biológicas, CSIC
Ramiro de Maeztu, 9
28040, Madrid, Spain
laraujo@cib.csic.es; j.m.andreu@cib.csic.es

† Present address: Max Planck Institute of Biochemistry, Martinsried, Germany

Supplementary Table S1. Chemical structures, MIC, MDIC and MDP values of FtsZ inhibitors.

Compound	Structure	MIC[g] (μM)		MDIC[g] (μM)	MDP[g] (min)
		B. subtilis	MRSA		
UCM62 (22)[a]	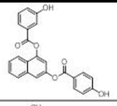	5	5	5	46
UCM78 (30)[a]	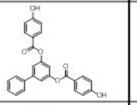	10	5	4	46
UCM79 (29)[a]	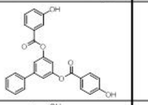	2.5	5	4	46
UCM81 (28)[a]	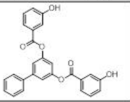	5	7	3.5	59
UCM82 (33)[a]	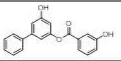	50	50	25	47
UCM93 (26)[a]	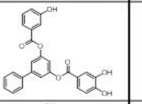	15	50	7.5	46
UCM95 (36)[a]	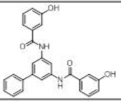	25	50	12.5	206
PC190723[b]	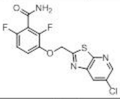	2.8	2.8	(5.6)	46
PC170942[c]	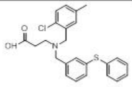	40	160	20	83
Hemi-chrysophaentin[d]	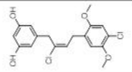	-	2	(18)	65
peptide MciZ[e]	MKVHRMPKGVVL VGKAWEIRAKLKEY GRTFQYVKDWISKP	-	-	1	47
Zantrin Z3[f]	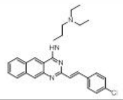	2.5	10	-	-

a. Artola, M., *et al.* (2015). ACS Chem Biol. 10: 834-843. Compound designation in the reference is shown in brackets.
b. Haydon, D.J. et al. (2008). Science 321: 1673-1675.
c. Stokes, N.R. *et al.* (2005). J Biol Chem. 280: 39709-39715.
d. Keffer, J.L., *et al.* (2013). Bioorg Med Chem 21: 5673-5678.
e. Bisson-Filho, A.W., *et al.* (2015). Proc Natl Acad Sci U S A 112: E2130-2138.
f. Margalit, D.N. *et al.* (2004). Proc Natl Acad Sci U S A. 101: 11821-11826.
g. MIC: minimal inhibitory concentration (references cited). MDIC: minimal division inhibitory concentration in *B. subtilis* (in brackets: division inhibitory concentration employed; see Methods). MDP: *B. subtilis* initial mass doubling period at MDIC. Control MDP value in the absence of inhibitors was 45 min.

Supplementary Table S2. Effect of PC190723 and its fragments on the assembly and GTPase activity of FtsZ polymers.

Measurements are average ± standard error. The critical concentration (Cr) values are the intercepts of the pellet FtsZ concentrations plots in Figure 3. The GTPase activities are the slopes of the GTP hydrolysis plots above Cr.

	**Bs-FtsZ**		**Ec-FtsZ**	
	Cr (μM)	GTPase (min^{-1}) (above Cr)	Cr (μM)	GTPase (min^{-1}) (above Cr)
**Control**	4.08 ± 0.06	2.17 ± 0.37	1.95 ± 0.06	3.87 ± 0.53
**PC190723 (15 μM)**	0.65 ± 0.13	0.22 ± 0.13	1.10 ± 0.18	5.30 ± 0.47
**DFMBA (4 mM)**	0.96 ± 0.02	0.40 ± 0.06	nd	nd
**CTPM (1 mM)**	3.85 ± 0.12	1.43 ± 0.16	nd	nd

Supplementary Table S3. Number of Z-ring per micron in control cell and cell treated with FtsZ inhibitors

	**Total Z-rings/Cell length (Z-ring/μm)** (Average ± SE)
**Control**	0.140 ± 0.006
**UCM81**	0.142 ± 0.010
**UCM93**	0.140 ± 0.023
**UCM95**	0.134 ± 0.014
**Hemi-chrys**	0.252 ± 0.016
**PC170942**	0.235 ± 0.013
**MciZ**	0.075 ± 0.014
**PC190723**	0.000 ± 0.000

Supplementary Table S4. Bacterial cell morphology measurements used in the PCA

	Cell length (μm)	Zrings/μm	Z*foci*/μm	Mb *foci*/ μm	Nucleoid length (μm)	Z-ring distribution (%)			
						0-2.5 (μm)	2.5-5 (μm)	5-7.5 (μm)	7.5-10 (μm)
**Control**	7.29 ± 1.12	0.14 ± 0.01	0.01 ± 0.01	0.11 ± 0.03	2.09 ± 0.04	0 ± 0.0	97 ± 0.5	2 ± 0.5	0 ± 0.0
**UCM81**	54.94 ± 4.35	0.14 ± 0.01	0.16 ± 0.02	0.53 ± 0.02	1.12 ± 0.09	47 ± 0.5	27 ± 0.5	17 ± 0.5	5 ± 0.1
**UCM93**	34.53 ± 2.42	0.14 ± 0.02	0.19 ± 0.04	0.05 ± 0.02	1.32 ± 0.12	35 ± 0.3	29 ± 0.4	32 ± 0.4	2 ± 0.9
**UCM95**	30.17± 1.69	0.13 ± 0.01	0.21 ± 0.04	0.04 ± 0.01	2.09 ± 0.13	12 ± 0.1	48 ± 0.5	36 ± 0.4	3 ± 0.3
**Hemi-chrys**	22.14 ± 1.53	0.25 ± 0.02	0.15 ± 0.01	0.38 ± 0.04	1.65 ± 0.10	36 ± 0.7	40 ± 0.8	18 ± 0.4	4 ± 0.1
**PC170923**	36.28 ± 3.31	0.23 ± 0.01	0.25 ± 0.04	0.16 ± 0.02	2.59 ± 0.06	31 ± 0.1	49 ± 0.2	18 ± 0.2	1 ± 0.6
**PC190742**	49.84 ± 6.61	0.00 ± 0.00	1.42 ± 0.06	0.36 ±0.05	2.69 ± 0.18	-	-	-	-
**MciZ**	41.04 ± 2.25	0.07 ± 0.01	0.06 ± 0.02	0.15 ±0.01	2.61 ± 0.16	0 ± 0.0	17 ± 0.9	20 ± 0.5	38 ± 0.5
**Nisin**	9.18 ± 0.60	0.18 ± 0.01	0.03 ± 0.01	0.14 ± 0.03	2.17 ± 0.04	2 ± 0.6	89 ± 0.5	7 ± 0.9	0 ± 0.0
**CCCP**	8.03 ± 1.04	0.00 ± 0.00	0.00 ± 0	0.31 ± 0.03	1.21 ± 0.06	-	-	-	-
**Kanamycin**	12.79 ± 0.93	0.04 ± 0.02	0.05 ± 0.02	0.44 ± 0.03	2.30 ± 0.19	-	-	-	-
**Cerulenin**	11.68 ± 1.20	0.23 ± 0.03	0.19 ± 0.04	0.09 ± 0.02	1.51 ± 0.10	24 ± 0.1	65 ± 0.5	10 ± 0.3	0 ± 0.0
**Daptomycin**	14.03 ± 1.07	0.12 ± 0.02	0.08 ± 0.01	0.46 ± 0.06	2.05 ± 0.08	2 ± 0.9	73 ± 0.5	20 ± 0.6	2 ± 0.9
**Vancomycin**	6.23± 0.33	0.04 ± 0.02	0.03 ± 0.02	0.34 ± 0.06	1.05 ± 0.07	-	-	-	-
**Cefotaxime**	17.92 ± 2.11	0.19 ± 0.01	0.09 ± 0.02	0.54 ± 0.06	1.54 ± 0.08	27 ± 0.3	73 ± 0.7	0 ± 0.0	0 ± 0.0
**Mitomycin C**	29.96 ± 2.37	0.17 ± 0.02	0.13 ± 0.03	0.60 ± 0.07	6.38 ± 0.67	9 ± 0.1	72 ± 0.7	18 ± 0.2	0 ± 0.0

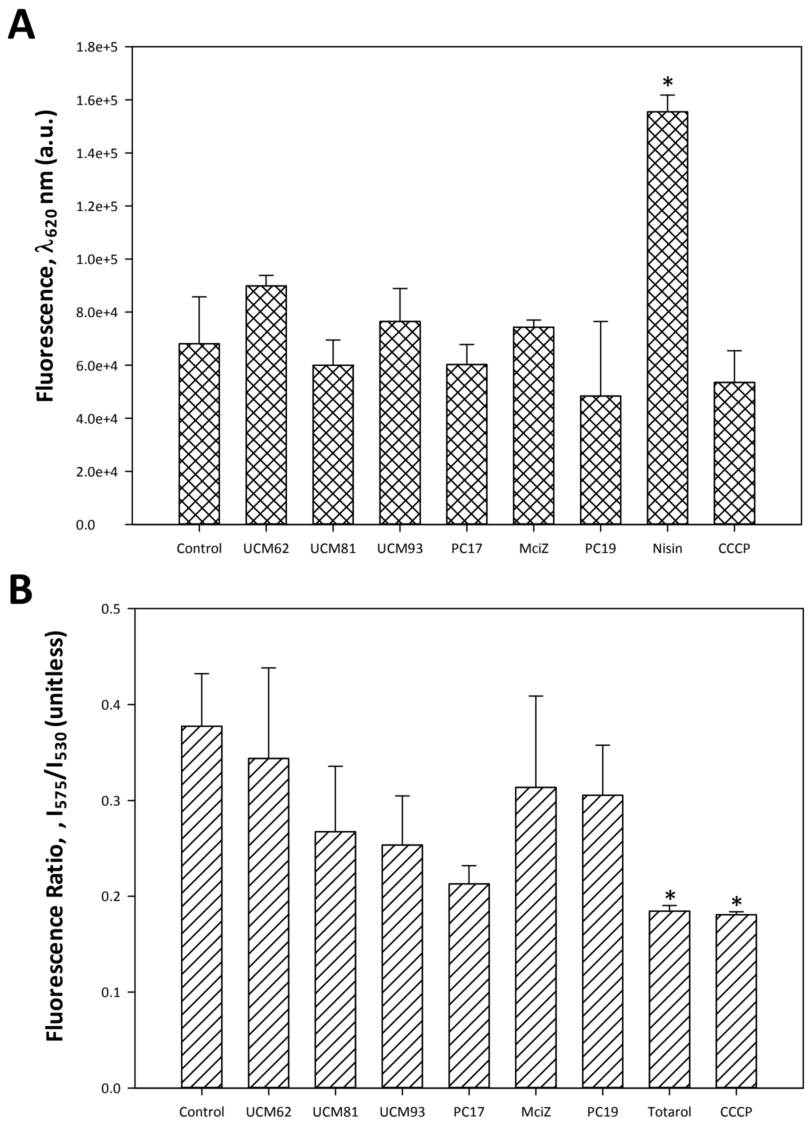

Supplementary Figure S1. Effect of FtsZ inhibitors on membrane permeability and membrane potential. (A) Fluorescence (620 nm) of *B. subtilis* 168 cells exposed to compounds and stained with PI were measured to assess membrane permeability. Significant differences ($p < 0.01$) were found only with the positive control compound nisin. **(B)** Fluorescence ratio of red (575 nm) to green (530 nm) fluorescence of *B. subtilis* 168 cells exposed to compounds and $DiOC_2$ were calculated to determined a possible membrane potential modification. Significant differences ($p < 0.01$) were found with totarol and CCCP (positive control).

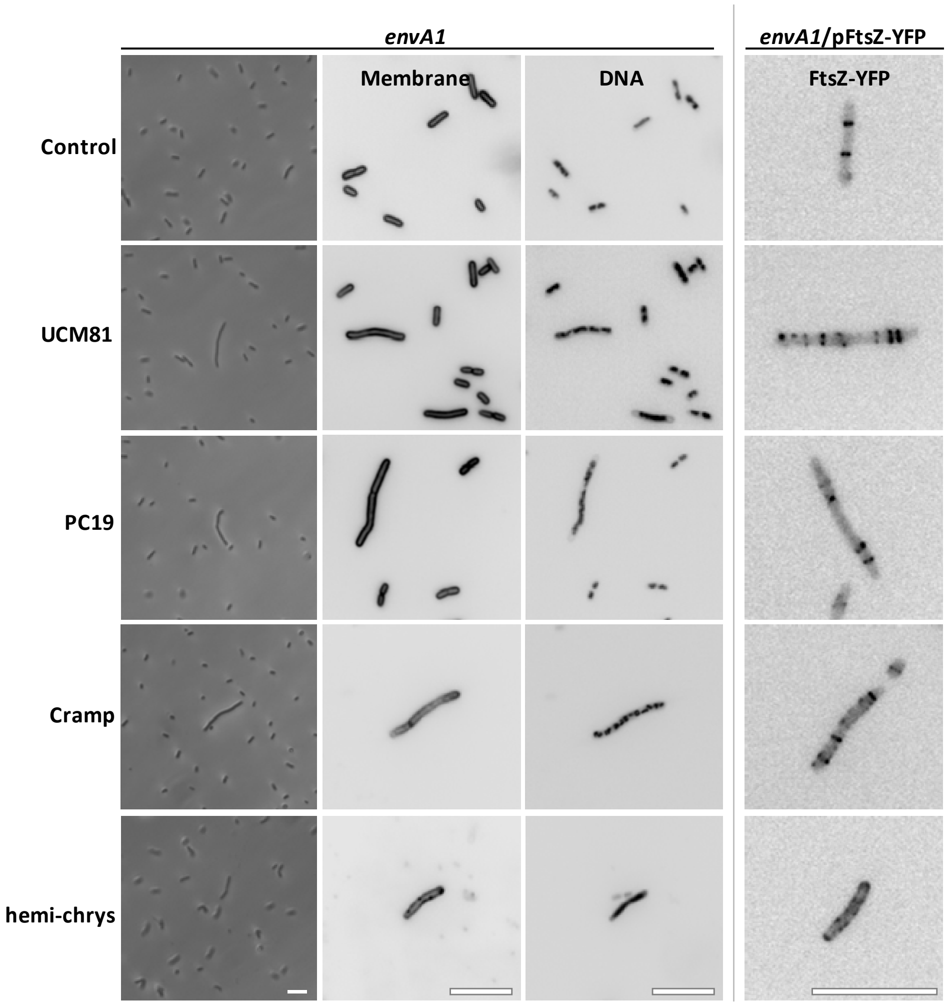

Supplementary Figure S2. Effects of FtsZ inhibitors on cell division, membrane, nucleoid and FtsZ subcellular localization in *E. coli* cells. Cells of *envA1 E. coli* strain were grown for 3 h in the presence of compounds, stained with FM4-64 and DAPI and visualized through their corresponding channels with a fluorescence microscope. For Z-rings analysis *envA1*/pFtsZ-YFP were used. After FtsZ-YFP induction cells were exposed to compounds for 1 hour prior to their analysis under the microscope. Images of the right column correspond to a general view of the sample but images of columns "Membrane", "DNA" and "FtsZ-YFP" show susceptible cells. Scale bar: 10 μm.

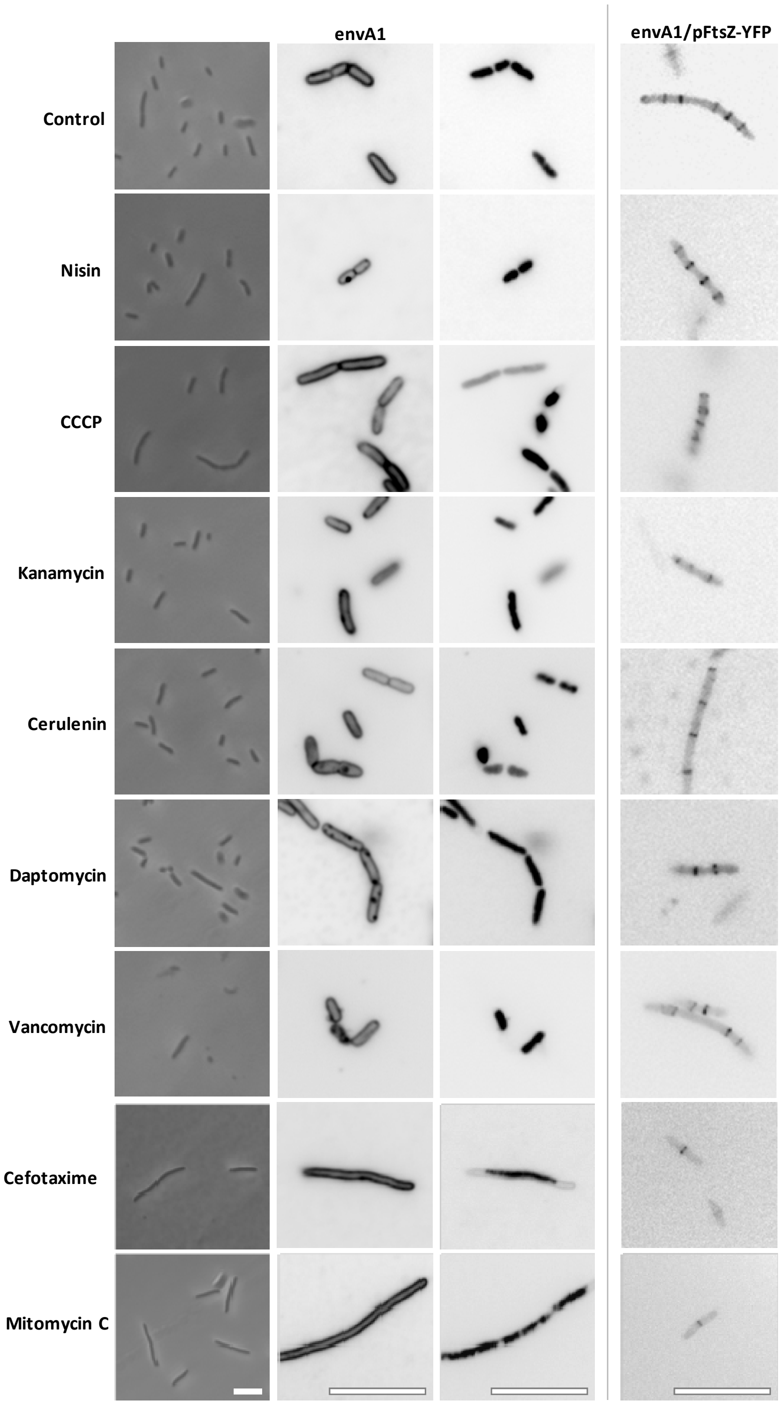

Supplementary Figure S3. Effect of antibiotics on membrane, nucleoid and FtsZ of *envA1* cells. Cells were incubated with compounds for 3 h and stained with FM4-64 to visualize the membrane and with DAPI to visualize nucleoids. For Z-rings analysis *envA1*/pFtsZ-YFP were used. After FtsZ-YFP induction cells were exposed to compounds for 1 hour and analyzed under the microscope. Scale bar: 10 μm.
